# Whole-Genome Sequencing Reveals Exonic Variation of *ASIC5* Gene Results in Recurrent Pregnancy Loss

**DOI:** 10.3389/fmed.2021.699672

**Published:** 2021-07-30

**Authors:** Nourah H. Al Qahtani, Sayed AbdulAzeez, Noor B. Almandil, Norah Fahad Alhur, Hind Saleh Alsuwat, Hatoon Ahmed Al Taifi, Ahlam A. Al-Ghamdi, B. Rabindran Jermy, Mohamed Abouelhoda, Shazia Subhani, Lubna Al Asoom, J. Francis Borgio

**Affiliations:** ^1^Department of Obstetrics and Gynaecology, College of Medicine, Imam Abdulrahman Bin Faisal University, Dammam, Saudi Arabia; ^2^Department of Genetic Research, Institute for Research and Medical Consultations, Imam Abdulrahman Bin Faisal University, Dammam, Saudi Arabia; ^3^Department of Clinical Pharmacy Research, Institute for Research and Medical Consultations, Imam Abdulrahman Bin Faisal University, Dammam, Saudi Arabia; ^4^Department of Nanomedicine Research, Institute for Research and Medical Consultations, Imam Abdulrahman Bin Faisal University, Dammam, Saudi Arabia; ^5^Saudi Human Genome Project, King Abdulaziz City for Science and Technology, Riyadh, Saudi Arabia; ^6^Department of Genetics, King Faisal Specialist Hospital and Research Center, Riyadh, Saudi Arabia; ^7^Department of Physiology, College of Medicine, Imam Abdulrahman Bin Faisal University, Dammam, Saudi Arabia; ^8^Department of Epidemic Diseases Research, Institute for Research and Medical Consultations, Imam Abdulrahman Bin Faisal University, Dammam, Saudi Arabia

**Keywords:** exome, recurrent pregnancy loss, whole genome sequencing, ASIC5, Saudi Arabia, molecular docking, next generation sequencing, unknown spontaneous abortion

## Abstract

Family trio next-generation sequencing-based variant analysis was done to identify the genomic reason on unexplained recurrent pregnancy loss (RPL). A family (dead fetus and parents) from Saudi Arabia with an earlier history of three unexplained RPLs at the ninth week of pregnancy was included in the study. Whole-genome sequencing (WGS) of a dead fetus and the parents was done to identify the pathogenic variation and confirmed through Sanger sequencing. WGS of dead fetus identifies a novel homozygous exonic variation (NM_017419.3:c.680G>T) in *ASIC5* (acid-sensing ion channel subunit family member 5) gene; the parents are heterozygous. Newly designed ARMS PCR followed by direct sequencing confirms the presence of heterozygous in one subject and absence of homozygous novel mutation among randomly selected healthy Saudis. The second family with heterozygous was confirmed with three unexplained RPLs. Pathogenicity analysis of R227I amino acid substitution in ASIC5 protein through molecular docking and interaction analysis revealed that the mutations are highly pathogenic, decrease the stability of the protein, and prevent binding of amiloride, which is an activator to open the acid-sensing ion channel of *ASIC5*. The identified rare and novel autosomal recessive mutation, c.680G>T:p.R227I (ASIC5^Saudi^), in two families confirm the *ASIC5* gene association with RPL and can be fatal to the fetus.

## Introduction

Recurrent pregnancy loss (RPL), or recurrent miscarriage (RM) is described as three or more sequential unpremeditated abortions before 20 weeks of gestation (1), a condition termed “habitual abortion” or “repeated spontaneous abortions” (2). RPL affects couples at propagative age around the world. The etiologies of RPL in Saudis or Arabs and other populations tend to be multifactorial. Factors including genetic abnormalities (3–10), placental anomalies (11–13), psychological trauma and stressful life events (14), and certain coagulation and immunoregulatory protein defects (15–18) were reported to be associated with RPL among women in the Gulf region. In some populations, other factors have been studied, such as anatomical, endocrine, hormonal problems, infection, smoking and alcohol consumption, and exposure to environmental factors, and these factors could increase the risk of RPL (19). Several studies have reported the relationship between various causes of recurrent miscarriage among Saudis and the rest of the population; however, 30–50% of RPLs were unexplained (5, 19). More studies on RPL only can reveal the cause. The objective of the study is to analyze the genetic basis of a family from Saudi Arabia with an earlier history of recurrent pregnancy loss at the ninth week of pregnancy using next-generation sequencing [whole-genome sequencing (WGS)] by complete analysis of whole genome of the fetus and parents followed by rigorous bioinformatics and confirmatory analyses (20–36). The study reports a novel homozygous exonic variation in the *ASIC5* gene in a dead fetus, while the parents are heterozygous.

## Materials and Methods

### Ethics and Study Subjects

The study was approved by the Institutional Review Boards Committee of the Imam Abdulrahman Bin Faisal University (IRB-2017-13-137).

A family with a past history of three miscarriages has been included in the study with a written consent from the father and mother. During the fourth pregnancy, the mother experienced a similar type of miscarriage at the ninth week of pregnancy. Tissue (separated cautiously from maternal tissue to avoid contamination) samples and blood samples were collected from the fetus (proband) and parents, respectively. Miscarriage sample was collected in an RNAprotect Cell Reagent (Qiagen, Hilden, Germany). The DNAs of the samples were isolated, and the most prevalent genetic disease, hemoglobinopathies, were screened using the Sanger sequencing. Genes (functional variants and deletions in *HBB, HBA1, HBA2, ATRX*, and *HBD*) related to the most prevalent mutations have been found to be normal. Hence, the WGS was done for the miscarriage tissue, mother, and father genomes.

### Whole-Genome Sequencing and Trio Analysis

The trio analysis has been carried out using the best practice GATK pipeline (20). The program Fastx (http://hannonlab.cshl.edu/fastx_toolkit) was used to filter low-quality reads. Then the reads were aligned to the reference human genome (hg19) using the program BWA (21). The GATK haplotype caller was used to call the variants. The resulting variants were then annotated using in-house developed workflow including the following three sets of data sources:

1. *Public databases*: These were collected from the Annovar packages, and they include the basic positional information about genes and related proteins. They also include information from the dbSNP database, the 1000 Genome database, ExAC, and gnomAD databases. Annovar also includes predictions of the functional effect of the variants from the tools Polyphan, Sift, CADD, and MetaSVM. In addition to Annovar, we used the clinvar and OMIM databases to annotate the variants and genes with up-to-data medical information.
2. *In-house databases*: We annotated the variants using the Saudi Human Genome Program variant DB to check for variant frequency in the Saudi population (22– 24).
3. *Commercial databases*: We used the HGMD database to annotate the variant with clinical information.

After variant annotation, we ran filters according to the ACMG (American College of Medical Genetics and Genomics) guidelines. We excluded variants that are intergenic, synonymous, appearing more than 5% in population databases, or not damaging (as predicted by CADD, Polyphen, SIFT, and MetaSVM). We also ran extra trio analysis to filter the variants according to the autosomal recessive, *de novo*, compound heterozygous, and x-linked. After applying these filters, the remaining variants were examined manually to match the annotated clinical information to the fetus phenotype.

### Sanger Sequencing Validation

Whole-genome result was confirmed using Sanger sequencing. The presence of the homozygous NM_017419.3:c.680G>T in the proband and heterozygous in the parents were confirmed using Sanger sequencing. Highly specific primers (*ASIC5*F: 5′-CAGATAAAAACATGTTTCCATACATCTTCAG-3′ and *ASIC5*R: 5′- TTGTGGCATGAACATTCCCTGGA-3′) were designed, and the selected region of the gene was amplified [PCR recipe: MOLEQULE-ON absolute master mix 12.5 μl, ASIC5F 1 μl (10 nM), ASIC5R 1 μl (10 nM), DNA Template 25 ng, and Dis H_2_O to 25 μl; temperature profile: 95°C for 10 min; 35 cycles of 95°C/60 s, 60°C/60 s, 72°C/60 s; and 72°C for 5 min] and sequenced using BigDye Terminator Cycle Sequencing Kit (Thermo Fisher Scientific, Inc., Waltham, MA, USA). Amplified PCR product (691 bp) of the *ASIC5* gene region was purified and sequenced using Genetic Analyzer 3500 (Thermo Fisher Scientific, Inc.) at the Department of Genetic Research, Institute for Research and Medical Consultations, Imam Abdulrahman Bin Faisal University (Dammam, Saudi Arabia). Sequences were analyzed using mutation surveyor software (Softgenetics, US) and DNA sequencing analysis software v.5.3 (Applied Biosystem; Thermo Fisher Scientific, Inc.).

### Amplification Refractory Mutation System-Polymerase Chain Reaction-Based Variation Screening and Sanger Sequencing Validation

The amplification refractory mutation system-polymerase chain reaction (ARMS-PCR) was designed (primers will be available on request) to screen the presence of NM_017419.3:c.680G>T among healthy Saudis (*n* = 200). The subjects positive for the presence of NM_017419.3:c.680G>T was confirmed through Sanger sequencing using primers (*ASIC5*F and *ASIC5*R). This is also to confirm the absence of the homozygous NM_017419.3:c.680G>T in the healthy Saudi subjects randomly selected.

### Homology Protein Modeling and Functional Annotations

The homology modeling of wild (p.R227) and mutant (p.R227I) ASIC5 protein was performed using Swiss Model server (25), validated using PROCHECK (26). The structural functional annotations were completed using SAS-sequence server (27), ProFunc (28), and PDBsum (29). Mutant structures were generated using Swiss-PDB Viewer and PyMol (30). Energy minimization for the wild and mutants was estimated using GROMACS (31). Evolutionary conservation and functional aspect analysis of the R227 residue in the wild-type protein was performed using the ConSurf (32). PROVEAN and I-Mutant were used for analyzing the impact on the biological function of a protein due to an amino acid substitution R227I (33, 34). AutoDock Vina was used for molecular docking of the ligand with wild type and mutant ASIC5 protein (35), and the molecular visualization was done in PyMol and LigPlot (36).

## Results

### Whole-Genome Sequencing and Trio Analysis

**Figure 1:**
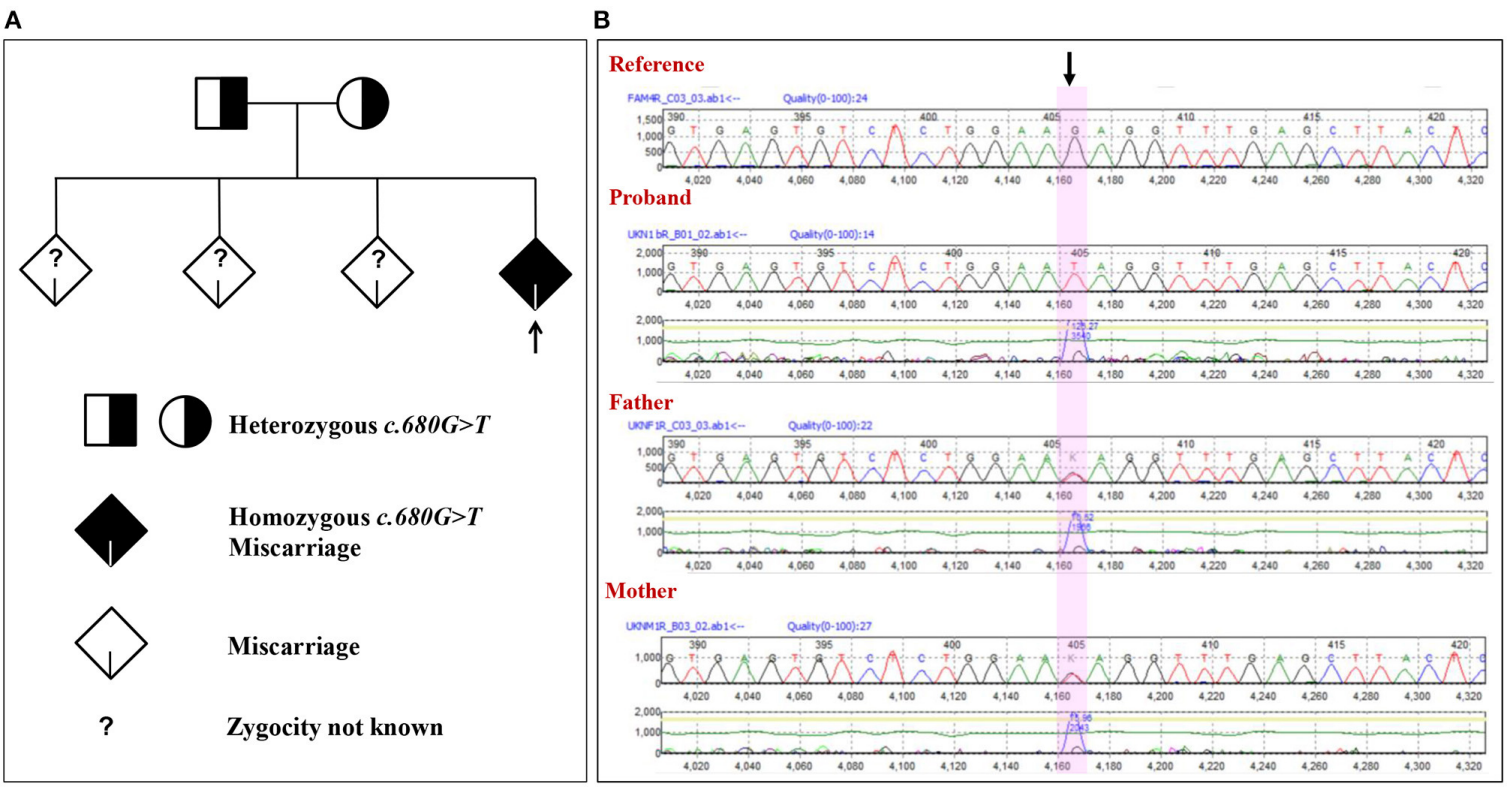
Novel mutation in the *ASIC5* gene (NM_017419.3:c.680G>T) in the family. **(A)** Phylogenic analysis of the family with the NM_017419.3:c.680G>T mutation in the *ASIC5* gene. **(B)** Electropherogram of the sequence c.664 to c.695 of exon 4 at the *ASIC5* gene of the proband and the parents. The highlighted nucleotide with arrow indicates the position of the NM_017419.3:c.680G>T. The proband is homozygous for the NM_017419.3:c.680G>T. The mother and father are heterozygous for the NM_017419.3:c.680G>T.

The family with a history of three unexplained miscarriages was included in the study. The couple is consanguineous but not first-degree relatives. There was no history of genetic and chronic diseases in the couple. The family was identified with a similar type of unknown spontaneous abortion at the ninth week of pregnancy. The mother was 30 years at the time of the fourth unexplained spontaneous miscarriage; the father was 34. The previous three unexplained miscarriages and the fourth were also of similar gestation. At this gestation, the gender of the proband cannot be determined even after miscarriage. The mother is devoid of uterine or cervical abnormalities. In order to identify the cause of the recurrent spontaneous abortion, WGS was done for the mother, father, and proband. The WGS of the trio (proband and parents) samples has revealed an inheritance of NM_017419.3:c.680G>T mutation in the *ASIC5* gene from the parents (Figure 1A and Supplementary Table 1). Various heterozygous mutations observed in the proband are listed in the Supplementary Material, which were inherited either from the mother or father (Supplementary Table 2). The WGS result of NM_017419.3:c.680G>T variation in exon 4 of the *ASIC5* gene has been confirmed through the Sanger sequencing (Figure 1B). The father and the mother were found to be carriers (heterozygous) of the c.680G>T:p.R227I at the *ASIC5* gene, while the proband was homozygous to c.680G>T:p.R227I (GenBank: MN251164; ClinVar: SCV000930628; SNP ID: rs1248841709) (Figure 1). The name of the novel variant was validated using Mutalyzer 2.0.32.

### Amplification Refractory Mutation System-Polymerase Chain Reaction-Based Variation Screening and Sanger Sequencing Validation

In order to confirm the absence of the homozygous NM_017419.3:c.680G>T among the living population, a total of 200 healthy Saudis were selected randomly and checked for the mutation at the c.680 position in the *ASIC5* gene using ARMS-PCR followed by Sanger sequencing. The results of the ARMS-PCR and direct sequencing of 200 healthy Saudis in the c.680 position in the *ASIC5* gene revealed the absence of homozygous NM_017419.3:c.680G>T. Furthermore, this mutation is novel to the SHGP (Saudi Human Genome Program) database (about 9,500 cases). This suggests that the discovered mutation NM_017419.3:c.680G>T is rare, and their absence of a homozygous state in the healthy Saudis is validated. Furthermore, a female subject was observed with a heterozygous NM_017419.3:c.680G>T in the *ASIC5* gene. The female subject with heterozygous mutations is a single daughter, and her mother experienced the unexplained RPL similar with the earlier family in the ninth week of pregnancy consecutively three times.

### Molecular Docking and Interaction Analysis

**Figure 2:**
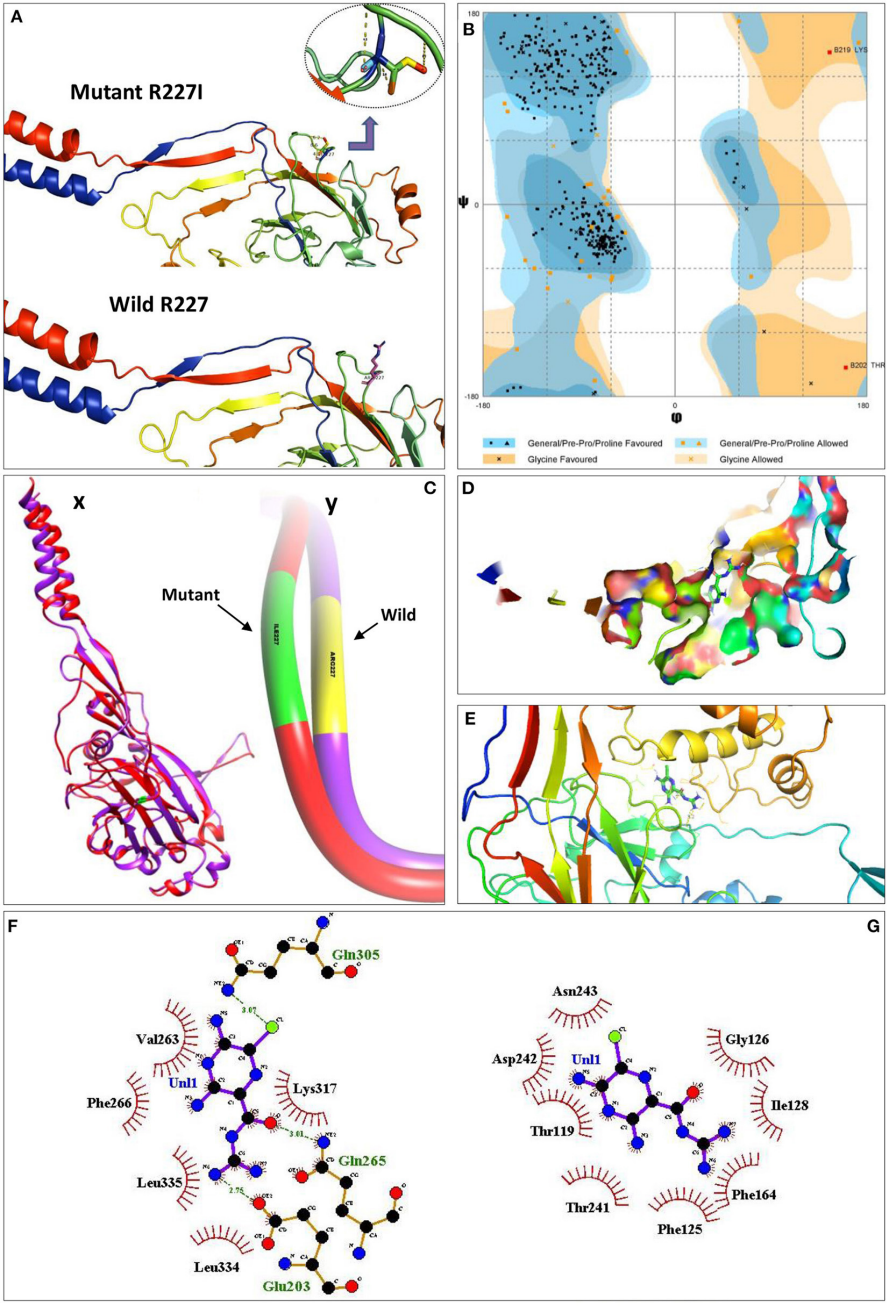
Pathogenicity analysis of R227I mutation in the ASIC5 protein through molecular docking and interaction analysis. **(A)** Structural models of the wild (R227) and mutated (R227I) ASIC5 proteins. **(B)** Ramachandran plot for the predicted structure of the ASIC5 protein. Eighty-three porterage residues of the ASIC5 protein are in the most favored regions. Cx, superimposed structures of the wild (R227) and mutated (R227I) ASIC5 proteins; Cy, deviated region of R227I from R227 on superimposed wild and mutant ASIC5. **(D)** Amiloride with ASIC5 at the active binding site. **(E)** 3D amiloride with surrounding amino acids of ASIC5 protein. **(F,G)** Protein–ligand interaction. **(F)** Wild ASIC5 (R227) protein with ligand, amiloride. **(G)** Mutant ASIC5 (R227I) protein with ligand, amiloride.

The predicted structure of the wild ASIC5 on the Ramachandran plot showed ϕ/Ψ angles of 83.1% residues in the most favored regions, 15.4% in the additional allowed regions, 1.1% in the generously allowed regions, and 0.3% in the disallowed regions (Figure 2B). The total residue span of the secondary structure consist of 23.0% residues involved in the formation of the strands, 23.0% residues in alpha helices, 2.6% residues in 3–10 helices, and 51.5% residues in other structural moieties. Analysis of secondary structure in ProFunc showed the presence of 3 β-sheets, 4 β-hairpins, 1 psi loop, 3 β-bulges, 14 strands, 14 helices, 5 helix–helix interactions, 34 β-turns, and 9 γ-turns. Homology modeling of the mutant structure (R227I) of the ASIC5 showed deviations from the wild type; the mutant structure on the Ramachandran plot showed ϕ/Ψ angles of 84.3% residues in the most favored regions, 14.3% in the additional allowed regions, 1.1% in the generously allowed regions, and 0.3% in the disallowed regions. The total residue span of the secondary mutant structure consisting of 22.7% residues involving the formation of the strands, 23.7% residues in alpha helices, 1.8% residues in 3–10 helices, and 51.8% residues in other structural moieties. Analysis of the secondary structure of the mutant in ProFunc showed the presence of 3 β-sheets, 4 β-hairpins, 1 psi loop, 2 β-bulges, 14 strands, 13 helices, 5 helix–helix interactions, 42 β-turns, and 8 γ-turns.

ConSurf analysis revealed that R227I is a functional residue, which is highly conserved and exposed. A total of 97 HMMER hits were considered for this analysis, while 91 of them were unique, including the query. PROVEAN analysis showed that R227I is a deleterious amino acid substitution as evident from PROVEAN score −3.830. I-Mutant analysis predicted that the free energy change value (DDG) between wild type and mutant type was less than zero (DDG < 0), which declares the decrease in protein stability. Wild (RMSD = 0.045 Å) and mutant (RMSD = 0.088 Å) proteins were superimposed, quantitative measure of similarity analysis revealed an increase of 95.56% root-mean-square deviation of atomic positions in the mutant (Figure 2C).

Molecular docking studies of wild (p.R227) and mutant (p.R227I) ASIC5 protein with amiloride, a potent inhibitor of acid-sensing ion channel proteins, were performed, and it was observed that the binding behavior of amiloride with the mutant model compared with the wild-type model was completely different (Figure 2). R227 residue is not directly involved in binding with the ligand, but it assists atomic interactions through binding of the ligand with protein molecules at specific sites (Figure 2F). In particular, a halogen bonding occurs between the chlorine atom (colored green) of amiloride with the amino group (NH_2_) of Gln305 (colored blue). The oxygen atom of the carbonyl group (colored red) of amiloride interacts with the hydrogen of the amino group (NH_2_) of Gln265 through N–H···O hydrogen bonding. In a similar fashion, the hydrogen of amiloride interacts with the oxygen group of Glu203. However, the R227I prevents the binding of ligand with the ASIC5 molecule at a specific site (Figure 2G). In this mutant model, an alteration in the protein coordination site occurs (Gly126 and Asn243) and, therefore, fails to coordinate with amiloride functional groups.

## Discussion

Studies on tissues of miscarriage specimens from women with RPL observed the chromosomal aberrations from 29 to 46% of miscarriage tissues, while majority of the RPL may be due to alternative mechanisms or other than chromosomal aberrations (37–39). The present observation suggests that coding variants in *ASIC5* gene can be one among the alternative mechanisms for RPL. The role of the acid-sensing ion channel subunit family member 5 (*ASIC5)* or *ACCN5* or bile acid-sensitive ion channel (*BASIC*) gene in humans, in general, and the development of the fetus, in particular, is scanty (40–42). Very limited studies are available on the gene *ASIC5* and related expression. This gene, *ASIC5*, was reported to be expressed in the amniotic fluid (43), fetal gut, brain, liver, heart, ovary, and testis (44). *ASIC5* is overexpressed in the fetal gut (41.0) and plasma (27.5). *ASIC5* was observed to a key player in the physiology of unipolar brush cells of the vestibulocerebellum (42, 45, 46). The complete functions of the *ASIC5* gene and its product are yet to be identified (40–42). Animal studies on the autosomal recessive mouse mutant of the gene encoding the L-type calcium channel revealed that the homozygous mutant animals die at birth; however, the heterozygous for the mutant is not distinguishable from that of wild animals (47). The study resembles the present observation of the heterozygous mutant of the healthy parents, while death of the fetus with a homozygous mutant in the gene belongs to the amiloride-sensitive Na^+^ channel. The R227I prevents the binding of amiloride with ASIC5 protein. However, more confirmatory studies are mandatory to prove the failure in amiloride-R227I (ASIC5) binding in wet lab, which is mandatory for an activator to open its own channel (41, 48). Acid-sensing ion channel subunit channels play an important role in the fetal developmental pathology due to acidosis; furthermore, prolonged acidosis is significantly associated with mortality of the fetus (49, 50). Increased apoptosis was observed in the retina due to the mutant *ASIC2* gene compared with the wild type (51). Mammalian degenerin (*MDEG*) or *ASIC2* (acid-sensing ion channel subunit 2) gene mutant study on the development of *Xenopus* reported that the *Xenopus* oocytes with *ASIC2* mutation start to maturate and die (52). This indicates the pathophysiology of the mutation in the acid-sensing ion channel subunit genes.

Earlier reports reported that in 39% of the Saudi females who had RPL, the origin of the patient in the study was unexplained or had no identifiable cause (5). Various reasons including genetic factors were stated for recurrent pregnancy loss among Saudi women (3, 4, 6, 14, 18). Consanguineous marriages are also considerably (*p* = 0.046) impacting (3). Genome-wide association study (GAWS) revealed the association of *ASIC5* (*p* = 0.0029; Supplementary Table 3) and level of manganese (53, 54). Furthermore, the level of manganese in the placental tissue of Saudi women with recurrent pregnancy loss was significantly (*p* < 0.0001) decreased (11). This suggests that the identified mutation in *ASIC5* might have played a role in the level of manganese in the present women. A recent study on the prognosis markers of glioblastoma revealed the expression of *ASIC5* as associated prognosis markers (55). *ASIC5* was found to be activated in the ethanol-(100 mM)-exposed neonatal rat cardiomyocytes along with other six molecules (*CYP2A6, PRL, CHRNA4, CNR1, CRH*, and *SLC40A1*) (56). Low (in 50%) ASIC5 protein expression in melanoma were observed with <4% mutation rates (57).

Preparing the mutated animal model to study the impact of the mutant on the fetal development is not available in our laboratory, which is a limitation of the study. Hence, the region with the mutation, c.680G>T in the *ASIC5* gene, was screened using ARMS-PCR followed by sequencing using designed primers to identify the presence of c.680G>T in randomly selected Saudis in the study region, which confirms the absence of the homozygous NM_017419.3:c.680G>T and the presence of heterozygous NM_017419.3:c.680G>T in a female subject and her mother with RPL. The study confirms the influence of the association of the novel exonic mutation with RPL. However, nationwide studies are mandatory to identify the prevalence of this rare mutation and mutations in this gene among unexplained miscarriages cases and their impact on the recurrent pregnancy loss and fetal development. This can reveal the role of *ASIC5*. The protein–protein interaction analysis of ASIC5 protein, with the protein observed with the mutation in the proband using STRING, revealed lack of interaction (Supplementary Table 2). However, the analysis using STRING cannot reveal any specific impact of mutated protein–protein interactions due to specific amino acid changes (58).

Based on the earlier reports on the member of the DEG/ENaC (degenerin/epithelial sodium channel) protein family and the current observations, it may be concluded that the R227I amino acid substitution in the *ASIC5* is highly deleterious; the mutant ASIC5 showed decreased stability and of the protein and prevents the binding of amiloride, a potent inhibitor of acid-sensing ion channel proteins (59).

The observed novel *ASIC5* gene-coding variant (ASIC5^Saudi^) in two families confirm the *ASIC5* association with the results of RPL. Hence, this mutation is pathogenic, which may cause serious illness to the fetus and cause fetal mortality. The molecular mechanism behind the death of the fetus in relation to the homozygous NM_017419.3:c.680G>T at exon 4 (ASIC5^Saudi^) in the *ASIC5* gene should be studied in detail. Early prenatal diagnosis of pathogenic variation like ASIC5^Saudi^ can provide a choice for the parent to decide pregnancy termination within the allowed time among high-consanguinity population (60).

## Data Availability Statement

The datasets presented in this study can be found in online repositories. The names of the repository/repositories and accession number(s) can be found in the article/Supplementary Material.

## Ethics Statement

The study was conducted in accordance with the Declaration of Helsinki and was approved by the Institutional Review Board (IRB) at Imam Abdulrahman Bin Faisal University. IRB approval number: IRB-2017-13-137 dated 07 June 2017 and extended on 14 Dec 2020. The patients/participants provided their written informed consent to participate in this study.

## Author Contributions

NHA, SA, NBA, HAA, AA-G, BR, and JB conceived and designed the research and analyzed the experiments. SA, NBA, NF, HSA, BR, and JB performed and analyzed the experiments. SA, MA, SS, and JB performed the whole-genome analysis. NHA, HAA, LA, and AA-G performed the clinical analysis. SA, HSA, and JB wrote the paper with the contributions of NHA, NBA, NF, HAA, AA-G, BR, MA, LA, and SS. All authors reviewed and approved the final manuscript.

## Conflict of Interest

The authors declare that the research was conducted in the absence of any commercial or financial relationships that could be construed as a potential conflict of interest.

## Publisher's Note

All claims expressed in this article are solely those of the authors and do not necessarily represent those of their affiliated organizations, or those of the publisher, the editors and the reviewers. Any product that may be evaluated in this article, or claim that may be made by its manufacturer, is not guaranteed or endorsed by the publisher.
