# Supplementary material for: Whole-Genome Sequencing Reveals Exonic Variation of ASIC5 Gene Results in Recurrent Pregnancy Loss
Source: Front Med (Lausanne). 2021 Jul 30;8:699672. doi: 10.3389/fmed.2021.699672 (PMC8363113; doi:10.3389/fmed.2021.699672)
Supplement: Supplementary file 1 [file Data_Sheet_1.PDF]

Whole genome sequencing reveals exonic variation of *ASIC5* gene results recurrent pregnancy loss

Nourah H Al Qahtani[1], Sayed AbdulAzeez[2], Noor B Almandil[3], Norah Fahad Alhur[2], Hind Saleh Alsuwat[2], Hatoon Ahmed Al Taifi[1], Ahlam A. Al-Ghamdi[1], B. Rabindran Jermy[4], Mohamed Abouelhoda[5,6], Shazia Subhani[5,6], Lubna Ibrahim Al Asoom[7], J. Francis Borgio[2,8*]

[1]Department of Obstetrics and Gynaecology, College of Medicine, Imam Abdulrahman Bin Faisal University, Dammam 31441, Saudi Arabia.

[2]Department of Genetic Research, Institute for Research and Medical Consultation (IRMC), Imam Abdulrahman Bin Faisal University, Dammam 31441, Saudi Arabia.

[3]Department of Clinical Pharmacy Research, Institute for Research and Medical Consultations (IRMC), Imam Abdulrahman Bin Faisal University, Dammam 31441, Saudi Arabia.

[4]Department of Nanomedicine Research, Institute for Research and Medical Consultations (IRMC), Imam Abdulrahman Bin Faisal University, Dammam 31441, Saudi Arabia.

[5]Saudi Human Genome Project, King Abdulaziz City for Science and Technology, Riyadh, Saudi Arabia.

[6]Department of Genetics, King Faisal Specialist Hospital and Research Center, Riyadh, KSA.

[7]Department of Physiology, College of Medicine, Imam Abdulrahman Bin Faisal University, P.O Box 2004, Dammam, 31541, Saudi Arabia

[8]Department of Epidemic Diseases Research, Institute for Research and Medical Consultations (IRMC), Imam Abdulrahman Bin Faisal University, Dammam 31441, Saudi Arabia.

S1 Table. **Autorecessive model analysis results of whole genome sequence of trio samples.**

**Features**	**Autorecessive model analysis results**
S_zyg	hom
M_zyg	het
F_zyg	het
Model	ARH
chromosome	chr4
start pos	156773374
end pos	156773374
context	GCTCAAACCTCTTCCAGAGAC
DP_Ref	0(Rfw=0)
DP_All	0(Afw=0)
reference	C
observed	A
quality	1488.77
filter	PASS
zygosity	hom
refGene function	exonic
refGene gene	ASIC5
refGene exonic function	nonsynonymous SNV

refGene AA change	ASIC5:NM_017419:exon4:c.680G>T:p.R227I
ensGene function	exonic
ensGene gene	ENSG00000256394
ensGene exonic function	nonsynonymous SNV
ensGene AA change	ENSG00000256394:ENST00000537611:<br>exon4:c.680G>T:p.R227I
knownGene function	exonic
knownGene gene	*ASIC5*
knownGene exonic function	nonsynonymous SNV
knownGene AA change	ASIC5:uc003ipe.1:exon4:c.680G>T:p.R227I
cytoBand	4q32.1
dbsnp	-
SGP777X2	0
SGP777X2HOM	0
SGP777X2(Exome)	0
SGP777X2(Exome)HOM	0
Screenee	2379
AC	0
AC_hom	0
SGPFreq	0

SGPFreqHom	0
SGP_AF_Strict	0
1000g2015aug_all	0
ExAC_pLI	2.26E-07
REVEL	0.326
Eigen	-0.053
SIFT_score	0.013
SIFT_converted_rankscore	0.538
SIFT_pred	D
Polyphen2_HDIV_score	0.239
Polyphen2_HDIV_rankscore	0.291
Polyphen2_HDIV_pred	B
Polyphen2_HVAR_score	0.348
Polyphen2_HVAR_rankscore	0.41
Polyphen2_HVAR_pred	B
MutationTaster_score	1
MutationTaster_converted_rankscore	0.588
MutationTaster_pred	D
MetaSVM_score	-0.641
MetaSVM_rankscore	0.631

MetaSVM_pred	T
M-CAP_score	0.051
M-CAP_rankscore	0.646
M-CAP_pred	D
CADD_raw	5.542
CADD_raw_rankscore	0.753
CADD_phred	26.4
GTEx_V6_gene	.
GTEx_V6_tissue	.
OMIM Gene Id	51802
OMIM type	Gene (*)
OMIM MimNumber	616693
OMIM Title	ACID-SENSING ION CHANNEL FAMILY MEMBER 5; ASIC5
OMIM Alt Titles	HUMAN INTESTINE SODIUM CHANNEL; HINAC
OMIM Comment	-
OMIM Disease Gene	ASIC5, HINAC
OMIM Disorders	Acid-sensing ion channel family member 5
OMIM Pred	H
OMIM Confidence	(C)onfirmed
OMIM Mapping Method	REc, H

VCF INFO	AC=2;AF=1.00;AN=2;BaseQRankSum=-8.540e-01;ClippingRankSum=-1.239e+00;DP=46;FS=0.000; GQ_MEAN=59.00;MLEAC=2;MLEAF=1.00;MQ=60.00;MQ0=0; MQRankSum=0.083;NCC=0;QD=33.08; ReadPosRankSum=0.303;SOR=0.836;VQSLOD=6.08;culprit=FS
VCF FORMAT key	GT:AD:DP:GQ:PL
VCF FORMAT value	1/1:2,43:45:59:1517,59,0

S2 Table. List of heterozygous mutations observed in the proband.

# Header = child	mother	father	other info (from child record if exists	otherwise from mother)								
**S_zyg**	**M_zyg**	**F_zyg**	**Model**	**Chromosome**	**start pos**	**end pos**	**context**	**DP_Ref**	**DP_All**	**reference**	**observed**
het	het	F_NA	NVL;NVR;CMP	chr20	2729253	2729253	TGCGGGGAATCCCAGAGACAT	0(Rfw=0)	0(Afw=0)	C	A
het	het	F_NA	NVL;NVR;CMP	chr20	2729254	2729254	GCGGGGAATCCCAGAGACATG	0(Rfw=0)	0(Afw=0)	C	T
het	het	F_NA	NVR;CMP	chr12	53343059	53343059	GCGCGGCCAGCGTCTATGCAG	0(Rfw=0)	0(Afw=0)	C	A
het	het	F_NA	NVR;CMP	chr12	53343069	53343069	CGTCTATGCAGGCGCTGGGGG	0(Rfw=0)	0(Afw=0)	G	T
het	het	F_NA	CMP	chr12	53343084	53343084	TGGGGGCTCTGGTTCCCGGAT	0(Rfw=0)	0(Afw=0)	G	C
het	M_NA	het	CMP	chr1	144830505	144830506	TATTCTTCGTGAGTGTGAGTGT	0(Rfw=0)	0(Afw=0)	GA	-
het	M_NA	het	CMP	chr1	144830511	144830512	TCGTGAGTGTGAGTGTGTGTGT	0(Rfw=0)	0(Afw=0)	GA	-
het	M_NA	het	CMP	chr9	97080923	97080925	AAGAGAGGTCGCTTCTTGGACTT	0(Rfw=0)	0(Afw=0)	GCT	-
het	M_NA	het	CMP	chr9	97080930	97080932	GTCGCTTCTTGGACTTGCTGGCA	0(Rfw=0)	0(Afw=0)	GGA	-
het	het	F_NA	CMP	chr12	40874774	40874774	ATCAACTGAAGGATCAGGGGC	0(Rfw=0)	0(Afw=0)	-	T
het	het	F_NA	CMP	chr12	40874777	40874777	AACTGAAGGATCAGGGGCAAC	0(Rfw=0)	0(Afw=0)	T	-
het	M_NA	het	CMP	chr14	94845825	94845826	GGGGTGCCTCCTCTGTGACCCC	0(Rfw=0)	0(Afw=0)	CT	-
het	M_NA	het	CMP	chr14	94845829	94845829	TGCCTCCTCTGTGACCCCGGA	0(Rfw=0)	0(Afw=0)	-	AC
het	het	F_NA	CMP	chr14	95566098	95566098	CACACACACACACACACACAC	0(Rfw=0)	0(Afw=0)	C	-
het	het	F_NA	CMP	chr14	95566100	95566108	CACACACACACACACACACA AACTTACCA	0(Rfw=0)	0(Afw=0)	CACA CACAC	-
het	het	F_NA	CMP	chr17	39254152	39254152	ACAGCAGGTGGGCTGGCAGCA	0(Rfw=0)	0(Afw=0)	-	A
het	het	F_NA	CMP	chr17	39254156	39254156	CAGGTGGGCTGGCAGCACACA	0(Rfw=0)	0(Afw=0)	G	-
het	het	F_NA	CMP	chr20	46279865	46279865	CAGCAGCAGCAACAGCAACAG	0(Rfw=0)	0(Afw=0)	-	GC
het	het	F_NA	CMP	chr20	46279866	46279866	AGCAGCAGCAACAGCAACAGC	0(Rfw=0)	0(Afw=0)	-	GCAG

S3 Table. Significant *ASIC5* gene annotations using Enrichr.

S. No	Data Base	**Name**	**P-value**	**Adjusted p-value**	**Z-score**	**Combined score**
1	KEGG 2019 Human	Inflammatory mediator regulation of TRP channels	0.005000	0.005000	-6.54	34.66
2	Reactome 2016	Stimuli-sensing channels_Homo sapiens_R-HSA-2672351	0.005000	0.01500	-1.92	10.16
		Ion channel transport_Homo sapiens_R-HSA-983712	0.01015	0.01523	-2.03	9.31
		Transmembrane transport of small molecules_Homo sapiens_R-HSA-382551	0.02970	0.02970	-2.11	7.44
3	Kinase Perturbations from GEO down	FGFR3_knockdown_174_ GSE41035	0.01500	0.01500	-1.95	8.17
		IGF1R_druginhibition_47_ GSE14024	0.01500	0.01500	-1.71	7.19
		ATM_knockout_16_ GDS1544	0.01500	0.01500	-1.70	7.16
		EGFR_druginhibition_82_ GSE27638	0.01500	0.01500	-1.66	6.99
4	GWAS Catalog 2019	Manganese levels	0.002900	0.002900	-1.57	9.15
5	DSigDB	NICKEL_CTD_00006389	0.009500	0.01900	-2.32	10.81
6	Tissue Protein Expression	HCC-202	0.01505	0.01720	-3.06	12.83

	from ProteomicsDB					
		BT-474 cell	0.01505	0.01720	-2.15	9.04
		CEM cell	0.01505	0.01720	-1.83	7.69
		BT-549 cell	0.01505	0.01720	-1.73	7.28
		NCI-H292 cell	0.01505	0.01720	-1.72	7.23
		CCRF-CEM cell	0.01505	0.01720	-1.71	7.18
		HCC-1937 cell	0.01505	0.01720	-1.63	6.85
