# Supplementary material for: Whole-Genome Sequencing Reveals Exonic Variation of ASIC5 Gene Results in Recurrent Pregnancy Loss
Source: Front Med (Lausanne). 2021 Jul 30;8:699672. doi: 10.3389/fmed.2021.699672 (PMC8363113; doi:10.3389/fmed.2021.699672)
Supplement: Supplementary file 2 [file Data_Sheet_2.PDF]

Name Checker

Please insert a variant description using the HGVS (http://varnomen.hgvs.org/) format.

Variant description

NM_017419.3:c.680G>T

Examples: AB026906.1:c.40_42del , NG_012337.1(SDHD_v001):c.274G>T , LRG_24t1:c.159dup

Check variant description

Help (https://github.com/mutalyzer/mutalyzer/wiki/Name-Checker)

0 Errors, 0 Warnings.

Overview of the raw variants

Raw variant 1: substitution at 731

```
AAGAGAAAAGTGAGTGTCTCTGGAA G AGGTTTGAGCTTACTCTTCAATGTG
AAGAGAAAAGTGAGTGTCTCTGGAA T AGGTTTGAGCTTACTCTTCAATGTG
```

Description relative to transcription start

(Not for use in LSDBs in case of protein-coding transcripts).

NM_017419.3:n.731G>T (/name-checker?description=NM_017419.3%3An.731G%3ET)

Affected transcripts - 1

NM_017419.3(ASIC5_v001):c.680G>T (/name-checker?description=NM_017419.3%28ASIC5_v001%29%3Ac.680G%3ET)

Affected proteins - 1

NM_017419.3(ASIC5_i001):p.(Arg227Ile)

Reference protein

```
  1  MEQTEKSKVY AENGLLEKIK LCLSKKPLPS PTERKKFDHD FAISTSFHGI HNIVQNRSKI
 61  RRVLWLVVVL GSVSLVTWQI YIRLLNYFTW PTTTSIEVQY VEKMEFPAVT FCNLNRFQTD
121  AVAKFGVIFF LWHIVSKVLH LQEITANSTG SREATDFAAS HQNFSIVEFI RNKGFYLNNS
181  TLLDCEFFGK PCSPKDFAHV FTEYGNCFTF NHGETLQAKR KVSVSGRGLS LLFNVNQEAF
241  TDNPALGFVD AGIIFVIHSP KKVPQFDGLG LLSPVGMHAR VTIRQVKTVH QEYPWGECNP
301  NIKLQNFSSY STSGCLKECK AQHIKKQCGC VPFLLPGYGI ECDLQKYFSC VSPVLDHIEF
361  KDLCTVGTHN SSCPVSCEEI EYPATISYSS FPSQKALKYL SKKLNQSRKY IRENLVKIEI
421  NYSDLNYKIT QQQKAVSVSE LLADLGGQLG LFCGASLITI IEIIEYLFTN FYWICIFFLL
481  KISEMTQWTP PPQNHLGNKN RIEEC*
```

Protein predicted from variant coding sequence

```
  1  MEQTEKSKVY AENGLLEKIK LCLSKKPLPS PTERKKFDHD FAISTSFHGI HNIVQNRSKI
 61  RRVLWLVVVL GSVSLVTWQI YIRLLNYFTW PTTTSIEVQY VEKMEFPAVT FCNLNRFQTD
121  AVAKFGVIFF LWHIVSKVLH LQEITANSTG SREATDFAAS HQNFSIVEFI RNKGFYLNNS
181  TLLDCEFFGK PCSPKDFAHV FTEYGNCFTF NHGETLQAKR KVSVSGIGLS LLFNVNQEAF
241  TDNPALGFVD AGIIFVIHSP KKVPQFDGLG LLSPVGMHAR VTIRQVKTVH QEYPWGECNP
301  NIKLQNFSSY STSGCLKECK AQHIKKQCGC VPFLLPGYGI ECDLQKYFSC VSPVLDHIEF
361  KDLCTVGTHN SSCPVSCEEI EYPATISYSS FPSQKALKYL SKKLNQSRKY IRENLVKIEI
421  NYSDLNYKIT QQQKAVSVSE LLADLGGQLG LFCGASLITI IEIIEYLFTN FYWICIFFLL
481  KISEMTQWTP PPQNHLGNKN RIEEC*
```

Effects on Restriction sites

Raw variant	Created	Deleted
1		MnlI

Experimental services

Genomic description: `731G>T`

Exon information

Number	Start (g.)	Stop (g.)	Start (c.)	Stop (c.)
1	1	91	-51	40
2	92	398	41	347
3	399	636	348	585
4	637	762	586	711
5	763	912	712	861
6	913	1060	862	1009
7	1061	1117	1010	1066
8	1118	1286	1067	1235
9	1287	1378	1236	1327
10	1379	1696	1328	*127

CDS information

	g.	c.
Start	52	1
Stop	1569	1518

Links

Download this reference sequence file: NM_017419.3.gb (/reference/NM_017419.3.gb)

Legend

Mutalyzer 2.0.32
released on 9 December 2019
Changelog (https://github.com/mutalyzer/mutalyzer/blob/master/CHANGES.rst)

HGVS nomenclature version 2.0 (notes (https://github.com/mutalyzer/mutalyzer/wiki/HGVS-Mutalyzer-Differences))
Recommended by (/about#recommended-by)

© 2009-2019 LUMC (http://www.lumc.nl)
Disclaimer (https://www.gnu.org/licenses/agpl-3.0.html)
